# Role of Lung Microbiome in Innate Immune Response Associated With Chronic Lung Diseases

**DOI:** 10.3389/fmed.2020.00554

**Published:** 2020-09-18

**Authors:** Keshav Raj Paudel, Vivek Dharwal, Vyoma K. Patel, Izabela Galvao, Ridhima Wadhwa, Vamshikrishna Malyla, Sj Sijie Shen, Kurtis F. Budden, Nicole G. Hansbro, Annalicia Vaughan, Ian A. Yang, Maija R J Kohonen-Corish, Mary Bebawy, Kamal Dua, Philip M. Hansbro

**Affiliations:** ^1^Centre for Inflammation, Centenary Institute, Sydney, NSW, Australia; ^2^Faculty of Science, University of Technology Sydney, Sydney, NSW, Australia; ^3^Discipline of Pharmacy, Graduate School of Health, University of Technology Sydney, Sydney, NSW, Australia; ^4^Priority Research Centre for Healthy Lungs, Hunter Medical Research Institute, The University of Newcastle, Newcastle, NSW, Australia; ^5^Faculty of Medicine, Thoracic Research Centre, The University of Queensland, Brisbane, QLD, Australia; ^6^Department of Thoracic Medicine, The Prince Charles Hospital, Brisbane, QLD, Australia; ^7^Woolcock Institute of Medical Research, University of Sydney, Sydney, NSW, Australia; ^8^School of Medicine, Western Sydney University, Sydney, NSW, Australia; ^9^St George and Sutherland Clinical School, University of New South Wales, Sydney, NSW, Australia

**Keywords:** asthma, chronic obstructive pulmonary disease, lung fibrosis, lung cancer, microbiome

## Abstract

Respiratory diseases such as asthma, chronic obstructive pulmonary disease (COPD), lung fibrosis, and lung cancer, pose a huge socio-economic burden on society and are one of the leading causes of death worldwide. In the past, culture-dependent techniques could not detect bacteria in the lungs, therefore the lungs were considered a sterile environment. However, the development of culture-independent techniques, particularly 16S rRNA sequencing, allowed for the detection of commensal microbes in the lung and with further investigation, their roles in disease have since emerged. In healthy individuals, the predominant commensal microbes are of phylum Firmicutes and Bacteroidetes, including those of the genera *Veillonella* and *Prevotella*. In contrast, pathogenic microbes (*Haemophilus, Streptococcus, Klebsiella, Pseudomonas*) are often associated with lung diseases. There is growing evidence that microbial metabolites, structural components, and toxins from pathogenic and opportunistic bacteria have the capacity to stimulate both innate and adaptive immune responses, and therefore can contribute to the pathogenesis of lung diseases. Here we review the multiple mechanisms that are altered by pathogenic microbiomes in asthma, COPD, lung cancer, and lung fibrosis. Furthermore, we focus on the recent exciting advancements in therapies that can be used to restore altered microbiomes in the lungs.

## Introduction

The lungs are vital organs that facilitate gas exchange during respiration. They are highly susceptible to disease risk factors such as exposure to air pollution, occupational exposure to toxins, cigarette smoke and infections. Hence, the burden of chronic lung diseases (CLDs) is rising (1–4). Lung diseases are the second leading cause of death worldwide (after cardiovascular disease) and account for more than 10% of disability-adjusted life years (5). Asthma, chronic obstructive pulmonary disease (COPD), idiopathic pulmonary fibrosis (IPF), and lung cancer are the most prevalent CLDs globally.

Briefly, asthma and COPD are chronic inflammatory diseases with different pathophysiology. The former is characterized by repeated episodic symptoms like shortness of breath and wheezing upon exposure to allergen, while the latter presents with chronic inflammation and irreversible airflow limitation (6–11). Around 334 million individuals suffer from asthma alone and the global prevalence of the disease has increased by 12.6% between 1990 and 2015 (12). Likewise, with a 44.2% increase in incidence, COPD is now the third leading cause of death worldwide (12, 13).

IPF is a progressive lung disease with a median survival rate of 3–5 years after diagnosis (14–16). Recent studies show that the rate of IPF is ~3–9 per 100,000 for Europe and North America, with limited data available on global prevalence (17, 18). It is characterized by progressive fibrosis of the lungs with an unknown cause leading to impaired forced expiratory volume, vital capacity and mortality in patients with an advanced stage of disease (19).

Lung cancer is one of the most commonly diagnosed cancers worldwide. Lung cancer is categorized into two major subtypes; 85% are non-small cell lung cancers (NSCLCs) and 15% are small cell lung cancers (20). Currently, lung cancer accounts for 18.4% of total cancer related deaths and therefore imparts a huge socio-economic burden on society (21). Furthermore, the prevalence of lung cancer is expected to rise due to the continued increase in risk factors such as smoking.

The causes of asthma, COPD, IPF and lung cancer are multifactorial and influenced by both genetics as well as the environment. Some of the known causes include long-term exposure to environmental and occupational hazards such as allergens, cigarette smoke, air pollution, asbestos, silica, coal dust, beryllium, hard metals, and radiation treatments (22, 23). These environmental factors along with genetic predisposition together drive the development and progression of lung disease.

Despite the increasing burden of these lung diseases, current treatment options are limited. In asthma, β2 agonists and inhaled corticosteroids remain as the first-line treatments, however 5–25% of patients fail to respond to steroids (6, 24, 25). Likewise, there is no effective therapy for COPD that stops or reverses the progressive nature of the disease and current disease management strategies primarily aim to treat associated symptoms (3, 26). For IPF, international guidelines for the management and treatment are lacking, with patients often limited to the prescription of anti-fibrotic drugs (nintedanib and pirfenidone) and broad spectrum antibiotics (azithromycin and polymyxin) (27, 28). For lung cancer, the overall 5-year survival rate was <5% for NSCLC until the last decade when the advent of immunotherapies raised this to 20–30% (29–31). Unfortunately, only 21% of NSCLC patients and 3.7% of small cell lung cancer patients are eligible for immunotherapy (32). Hence, the development of better treatment strategies is urgently needed, which can be achieved through improved understanding of the pathogenesis of CLDs.

There is growing interest in understanding host-microbiome interactions and manipulating the lung microbiome by targeting specific microbes or microbial products such as toxins and metabolites as potential novel treatments. There have been major recent advances in the understanding of the molecular and cellular mechanisms of CLDs, and new research is continuously revealing that many of these processes are driven by interactions with the respiratory microbiome (6, 33, 34). Metagenomics is the investigation of the collective genomic material in an environment (35). DNA libraries are prepared from samples such as bronchoalveolar lavage fluid (BALF) and tracheal aspirates, sequenced using Miseq (next generation sequencing technology) and analyzed using metagenomics tools. This approach provides a broad scale analysis of microbial species, their distribution, metabolic properties and impact on disease. Several factors influence bacterial persistence in the lower respiratory tract including; oxygen gradients, nutrient availability, temperature, pH and surfactants such as phosphatidylcholine-containing lipids (primarily dipalmitoyl phosphatidylcholine) and sphingomyelins (36). Furthermore, metagenomics can be used for molecular diagnostics and surveillance of pathogens in diseases (37).

One of the key characteristics used to assess the microbiome is diversity: the number of different species (richness) and the abundance of different species (evenness). Diversity of bacterial species can be measured within samples, which is known as α-diversity (using Simpson or Shannon index) or between samples, which is known as β-diversity (using principal coordinate analysis) (38). Bacterial taxa found in healthy individuals include Bacteroidetes and *Firmicutes* comprising of *Veillonella, Lachnospira*, and *Rothia* that have been detected in nasopharyngeal swabs, oral washes or bronchoalveolar lavage (39). A microbial imbalance, known as dysbiosis, has been associated with the development of several diseases including lung diseases (40).

Recent studies have highlighted the modulatory role of the microbiome in inflammation and inflammatory diseases. The term “microbiome” refers to the aggregate of all the commensal microbiota present on or in a particular host, including viruses, fungi, bacteria, archaea, and protozoa (1). The human microbiome is highly diverse, with distinct microbial profiles at different anatomical sites, and has been implicated in disease. For instance, it has been well established that that microbiome in the gut plays a distinct role in the pathogenesis of diseases such as irritable bowel syndrome, ulcerative colitis, allergy (e.g., atopic dermatitis) and obesity (41–45). Accordingly, recent research suggests that the resident lung microbiome is altered during the development of lung diseases, which allows for the colonization of pathogenic bacteria. Several bacterial species such as *Legionella* (46), *Escherichia coli, H. influenzae, S. pneumoniae, Enterobacter* spp. (47), and *Moraxella* spp. (48) can initiate lung inflammation. Until recently, the lungs of healthy individuals were considered to be sterile as culture-dependent techniques were unable to detect the presence of bacteria. However, with the development of culture-independent techniques, microbes of different phyla and genera have been found in the lungs and are associated with lung disease (49). The alteration in respiratory microbiomes during CLDs and how they are affected by challenges such as indoor/outdoor air pollution, biomass/cigarette smoke, and pathogenic infections are being recently elucidated. The immunological crosstalk between gut and lung, termed as “gut-lung axis” can impact the health of the lung as microbiome in lung are altered in CLDs. This involve activation of numerous factor such as inflammasomes and microRNAs (4). It is now well-established that the primary microbiome phyla residing in healthy lungs include *Firmicutes, Bacteroidetes, Proteobacteria, Actinobacteria* and others (49). Pathogenic microbes, such as *Pseudomonas aeruginosa* and *Haemophilus influenzae*, have the capacity to trigger different immune responses. Thus, it may be beneficial to examine the role of an altered lung microbiome in the pathogenesis and progression of lung diseases (1). Accordingly, in this review, we will discuss recent updates regarding the role of the lung microbiome in the context of COPD, asthma, IPF and lung cancer.

## Lung Microbiome and Asthma

Asthma affects >330 million people worldwide and is a major healthcare issue. The annual increase in asthma incidence was 1.4% for children and 2.1% for adults between 2011 and 2015 (24, 50). It is a complex and heterogeneous inflammatory disease with variable phenotypes. Classically, asthma is characterized by increased type 2 immune responses and eosinophilic inflammation (51). However, recent studies have linked elevated type 1/17 immune responses with non-eosinophilic (i.e., neutrophilic) inflammation especially in adults with moderate to severe asthma (24, 51, 52). Interestingly, exogenous factors including respiratory viral and bacterial infections, a high-fat diet and/or obesity, air pollution and cigarette smoke exposure are associated with severe asthma in adults as well as with exacerbations of the disease (24, 53–56).

Unfortunately, 5–10% of asthmatics do not respond to steroid therapies and are more likely to have severe disease. Severe steroid-resistant (SSR) asthma is a condition where asthmatic patients do not respond to mainstay corticosteroid therapies (57). SSR is associated with non-eosinophilic endotypes of the disease, including neutrophilic asthma, and involves activation of innate immune responses, particularly those mediated by Toll-like receptor (TLR)2 and TLR4 responses, and NLRP3 (nucleotide-binding oligomerisation domain-like receptor family, pyrin domain-containing 3) inflammasome/interleukin (IL)-1β responses (11, 25, 56, 58–60). The lung microbiome has been linked SSR asthma. Recently, Durack et al., showed that variations in bronchial microbiome composition is associated with the immunological, steroid-responsiveness and clinical features of asthma. Patients with asthma had an enrichment of Haemophilus, Neisseria, Fusobacterium, Porphyromonas and Sphingomonodaceae, and a depletion of Mogibacteriaceae and Lactobacillales. The study also showed distinct changes in specific microbial members after fluticasone treatment, demonstrating that steroid treatment can alter the microbiome which may influence steroid-responsiveness (61). Accordingly, researchers have shown that pathogenic microbes such as *Haemophilus* spp. and *Chlamydia* infections induce the development of SSR experimental asthma when combined with mild to moderate asthma models (62–67). Therefore, a thorough understanding of the lung microbiome may be essential to understand the immunological differences between asthma endotypes and how to treat them.

Several studies have characterized the bacterial profile of the lung microbiome in asthma. In asthma there is an elevated abundance of *Haemophilus influenzae, Streptococcus pneumoniae, Staphylococcus aureus*, and *Moraxella catarrhalis* in nasopharyngeal swabs, compared to healthy controls. While frequently detected as part of a normal microbiome, these bacteria are known pathogens that can cause infectious exacerbations (35). Furthermore, across two studies, Huang et al., showed that *Actinobacteria* can have a high abundance in patients with severe asthma, which correlate with disease outcomes, including elevated sputum leukocytes and eosinophils in bronchial biopsies (68), and the abundance of *Comamonadaceae, Sphingomonadaceae*, and *Oxalobacteraceae* in asthma patients correlates with airway hyperreactivity (69). Denner et al., showed that bacterial α-diversity in endobronchial brushings of asthmatic subjects corresponds with eosinophil numbers in lavage as well as forced expiratory volume in 1 s (FEV_1_) (70). The alterations in lower airway microbiomes of patients with asthma also correlated with FEV_1_ as a biomarker of airflow obstruction. Patient with asthma with FEV_1_ < 60% had low α-diversity but high β-diversity, low abundance of *Firmicutes, Bacteroidetes*, and *Actinobacteria* and potential pathogenic genus Streptococcus and the commensal genera Veillonella and Prevotella, compared to asthma patients with an FEV_1_ > 80% (70). Overall, these studies demonstrate that the lung microbiome has an impact on patient outcomes in asthma.

In addition to patient outcomes, the lung microbiome is associated with systemic and bronchial markers of inflammation (71). In the lower airway microbiome of patient with severe asthma, pathogenic microbes *Moraxella catarrhalis, Haemophilus* spp., and *Streptococcus* spp. are predominate and their presence correlates with cytokine levels (IL-8), eosinophilia, and neutrophilia (72, 73). *S. pneumoniae* is another pathogenic microbe that is associated with asthma, but conflicting reports are available regarding its influence. In a mouse model of allergic airway disease, it was reported that mice infected with non-lethal *S. pneumoniae* as neonates before sensitization and challenge with ovalbumin (OVA) had increased interleukin-17A (IL-17A) production, elevated neutrophil recruitment and Th17 cells (74). However, we previously showed that exposure to non-pathogenic *S. pneumoniae*, through either infection, killed bacteria or its components, can protect against allergic airway disease in adult mice through the modulation of dendritic cells, natural killer cells, and the induction of regulatory T cells (Tregs) (75–79). It would be interesting to continue this work and examine the role of microbial dysbiosis on the development of SSR asthma.

Microbial metabolites, such as short chain fatty acids (SCFAs) acetate, propionate and butyrate, regulate the physiology and immune response in humans (80). SCFAs are produced by the gut microbiome via fermentation of dietary fibers and are distributed across the body through the bloodstream. SCFAs are utilized by the body to provide energy or as signaling molecules (81, 82). SCFAs inhibit histone deacetylases (HDACs) and promote anti-inflammatory cell phenotypes (i.e., neutrophils) that maintain homeostasis, and suppress nuclear factor kappa-light-chain-enhancer of activated B cells (NF-κB) and tumor necrosis factor-α (TNF-α) production (80, 83, 84). Cait et al., identified SCFAs and the depletion of SCFA-producing bacteria as a mechanistic link between the microbiome and asthma susceptibility or severity. Exacerbation of OVA-induced allergic lung inflammation by vancomycin treatment was due to the lack of gut microbiome populations responsible for producing SCFAs, particularly butyrate. This work highlighted the potential of probiotics as a novel therapy for asthma (85). Hougee et al., utilizing OVA induced mouse model of asthma reported that oral administration of bacteria *Bifidobacterium breve* M-16V and *Lactobacillus plantarum* NumRes8 reduced the eosinophilic inflammation, OVA-specific IgE levels, and cytokines production (86). Likewise, the protective effects of administration of *Lactobacillus rhamnosus* GG and *Lactobacillus paracasei* L9 have been reported against the birch pollen-induced allergic asthma and urban airborne particular matter 2.5 μm-induced enhancement of airway hyperresponsiveness in the mouse model of asthma (87, 88). Huang et al., evaluated the effect of *Lactobacillus* aracasei, Lactobacillus fermentum or their combination on immune biomarkers, clinical severity and quality of life in 160 children aged 6–18 years of age with asthma in a double-blind, prospective, randomized, placebo-controlled trial. Serum IFN-γ, IL-4, IgE and TNF-α levels were evaluated for immune biomarkers, but only levels of IgE decreased significantly in the combination treatment group. Combined *L*. aracasei and L. fermentum also significantly improved quality of life. All intervention groups had lower levels of Clostridium compared to controls (89). This suggests that colonization by other microorganisms, and increasing gut microbial diversity, are potentially other protective factors against asthma (90). However, further studies are required to better understand the role of the microbiome in asthma and to define the potential for therapeutic manipulation.

## Lung Microbiome and COPD

The prevalence of COPD is increasing, and its management is a formidable challenge to healthcare systems. There is growing evidence that the lung microbiome is altered during the course of the disease and contributes to COPD pathogenesis (91–93). Hilty et al., provided early evidence of the presence of dysbiosis in COPD. 16S rRNA analysis was performed on DNA collected from swabs from the nose and oropharynx, and bronchial brushings from the left upper lung lobe of COPD patients and healthy controls. Sequencing showed that COPD patients have a distinct microbiome in their lungs compared to healthy individuals. Specifically, pathogenic *Proteobacteria* (*Haemophilus* spp.) were more common in COPD than *Bacteroidetes*, with *Prevotella spp*. being especially reduced (94). Subsequent studies showed the predominance of *Firmicutes, Bacteriodetes, Proteobacteria, Fusobacteria*, and *Actinobacteria* in healthy individuals, in contrast to pathogenic *Haemophilus, Streptococcus, Klebsiella, Pseudomonas, and Moraxella* in COPD patients (91–93, 95, 96). We propose that the lung microbiome is dynamic and transient, with specific taxa being present at different stages of disease progression and impacted by clinical factors such as age, smoking, medications, or seasonal changes (33, 34).

Cigarette smoking is a major risk factor for many lung diseases. Hence, researchers have investigated the effects on the lung microbiome. A growing number of reports indicate that smoking alters the oral and respiratory bacterial microbiome (97–99). Limited studies have been carried out in the context of COPD. Erb-Downward et al., assessed BALF samples from smokers with COPD, healthy smokers and non-smokers and showed notable β-diversity (i.e., differences in bacterial evenness) between the cohorts which is likely caused by outgrowth of bacteria within the population, rather than colonization of different bacteria (91). However, contradictory results were found in a subsequent study by Sze et al., utilizing lung tissue samples from non-smokers, smokers without COPD, and COPD patients where there was low diversity between non-smokers and healthy smokers, and a high β-diversity compared to COPD (smokers & non-smokers) (100). The disparity in outcomes may be due to differences in samples used (BALF vs. lung tissue) and small sample size. Nevertheless, these studies suggest that cigarette smoke is associated with alterations in lung bacterial microbiomes in healthy smokers and COPD patients. Whether these changes in bacterial populations are associated with the development and progression of COPD still needs to be determined.

In addition to the impact of cigarette smoke on bacterial populations, a recent study reported that cigarette smoke alters the lung virome. In BALF samples from smokers and non-smokers, bacterial communities were found to be similar among the two groups; however, significant differences were found in the lung virome, which correlated with levels of IL-8 and arachidonic acid. Both IL-8 and arachidonic acid are involved in COPD pathogenesis. The former is a potent neutrophil attractant and the pro-inflammatory effects of the latter have been reported in COPD (101, 102). In smokers, bacteriophages infecting *Prevotella, Actinomyces, Aeromonas, Capnocytophaga, Haemophilus, Rodoferax, and Xanthomonas* were increased, while *Enhydrobacter* and *Morganella* bacteriophages were dominated in non-smokers (103). Future studies are required to address: (1) which microbiome populations (bacterial, viral, fungal) in the lung are influenced by smoking, and; (2) how changes in the microbiome contribute to the susceptibility and progression of COPD.

Furthermore, smoking can indirectly effect the lung microbiome by altering the extra-cellular matrix (ECM) (104–107). *H. influenzae, S. pneumoniae, Pseudomonas*, and *Moraxella* are frequent pathogenic colonizers in COPD patients (108). During non-typeable *H. influenzae* (NTHi) infections, the ECM proteins fibronectin, laminin, collagen IV, and vitronectin play critical roles in colonization. Briefly, NTHi binds to the host vitronectin via bacterial protein E resulting in subsequent intracellular invasion. Further, high affinity of Hap (one of the bacterial adhesins) has been reported for fibronectin, laminin, and collagen IV, which eventually aid NTHi invasion (109, 110). Likewise, during lung infections by *Streptococcus, Pseudomonas*, and *Moraxella*, laminin, collagen I, V, and VI are involved in adhesion and invasion (111). Since the levels of ECM proteins (fibronectin/fibrinogen/collagen/laminins/fibulin-1c) are altered in patients with COPD, relationship between smoking, pathogenic microbes and ECM need to be explored (107, 112–114).

Research investigating the mechanism by which the lung microbiome can influence the COPD-linked immune response is in its infancy. Studies have shown pathogenic microbes cause lung inflammation in healthy individuals (115–118). The immune-stimulatory capacity of Gram-negative pathogenic bacteria associated with COPD (Haemophilus spp. & Moraxella) is significantly higher than Gram-negative commensal bacteria (Prevotella spp.) (119). Further, poor oral health is an important risk factor for inflammatory lung diseases (120). Studies have shown that microaspiration of oral taxa (particularly *Veillonella* and *Prevotellla*) is associated with elevated Th17 lymphocytes in lungs (115, 121). Thus, dysbiosis in the COPD lung may be associated with COPD exacerbations (122). Epithelial cells and alveolar macrophages form the first line of defense against pathogens. They express pattern recognition receptors that recognize pathogen-associated molecular patterns to initiate the innate immune response. *H. influenzae* interacts with TLR2 and activates NF-κB (the master regulator of pro-inflammatory response) via translocation dependent and independent pathways. In the translocation dependent pathway, activation of NF-κB inducing kinase (NIK)–IKKα/β complex leads to KiBα phosphorylation and there is degradation. The NF-κB translocation-independent pathway occurs through activation of MKK3/6–p38 mitogen-activated protein (MAP) kinase pathway (123, 124). In addition to TLR2, TLR4 induced NF- κB activation has also been reported in context of *H. influenzae* (125, 126). Like *H. influenzae, P. aeruginosa* is recognized by pattern recognition receptors. *P. aeruginosa* activates NF-κB via TLRs through both TIRAP-dependent and independent pathways. Additionally, this bacterium is involved in the activation of MAPK cascades (127). Thus, pathogenic microbes induce inflammatory responses that are part of the natural defense against foreign pathogens. However, in COPD patients, excessive, persistent and chronic inflammation is a major driver of disease pathogenesis. The activation of TLRs by the presence of pathogenic microbes can further aggravate the inflammatory state in the lungs of COPD patients (128). This eventually results in tissue damage and the production of damage-associated molecular patterns that further activate inflammatory pathways, leading to a vicious cycle of pathogenesis (129). Our recent study utilizing TLR2- and TLR4-deficient mice showed that TLR2 and TLR4 play opposing roles in COPD pathogenesis. TLR2 inhibition increased COPD features in an experimental mouse model, while these features were reduced in the absence of TLR4 (128). Thus, modulation of TLRs appear to be a potential strategy to suppress COPD and bacteria-induced exacerbations. However, future studies examining the relationship between TLRs expression, bacterial load, and lung inflammation area required. Further, the protective role of TLR-2 and 4 through activation of innate immune system should not be ignored.

Additionally, studies have been carried out for the analysis of microbiome dynamics associated with COPD exacerbations. Wang et al., and Mayheve et al., utilizing sputum samples from the COPD subjects reported that *Moraxella* abundance is increased during COPD exacerbation compared to stable COPD subjects. However, *Haemophilus* abundance did not show significant change between stable and exacerbated COPD individuals. Both *Haemophilus* and *Moraxella* are known to form biofilms that protect them from antibiotics and immune cells, thus their persistence is associated with repeated bacterial exacerbations (122, 130). It is important to mention here that different sampling procedures like BALF, sputum, lung tissue explants represent microbiome from different respiratory regions. Hence, future studies utilizing BALF and lung tissue samples should be carried out to study microbiome dynamics with different stages of COPD. Further, thresholds should be determined to identify significant bacterial over-representations for all sample types.

Thus, a limited number of studies have explored the role of lung dysbiosis in COPD but it is difficult to draw any conclusions. Although they are valuable for our understanding, there are issues with existing studies such as the use of different samples (BALF vs. lung tissues), limited numbers of participants, low biomass and potential for contamination. Further studies are required to elucidate the role of the lung microbiome in the pathogenesis and progression of COPD. Additionally, studies evaluating the impact of TLR-agonists should be performed.

## Lung Microbiome and Lung Cancer

The main causative factors in lung cancer are exposure to carcinogens, cigarette smoke, toxic compounds in the environment/industry, chronic airway inflammation driven by pathogenic infections, and fibrosis/scarring from co-morbid lung disease (30, 131). The lung microbiome may be altered by these causative factors and has been linked to lung cancer progression, phenotype and severity.

Research has shown that specific species/genera/phyla of bacteria are associated with lung cancer progression; specifically, *Helicobacter pylori* (132)*, Acidovorax temporans* (133), Cyanobacteria (134), Actinobacteria, Bacteroidetes, Proteobacteria, Firmicutes (135)*, Cytomegalovirus* (136)*, Thermus, Legionella, Megasphaera, Veillonella* (137), Capnocytophaga, Neisseria and Selenomonas (138). The specific pathogenic mechanisms of microbiome-mediated lung cancer progression are not widely known include the effects of bacterial toxins such as lipopolysaccharide (LPS) and inflammatory cytokine release by immune cells. The LPS of *H. pylori* can stimulate the production of pro-inflammatory factors including TNF-α, IL-1, and IL-6. These inflammatory mediators promote chronic lung diseases such as COPD and bronchitis that are followed by lung cancer (133). Various other bacterial toxins also have crucial roles in tumor initiation and progression. Cytolethal distending toxin (CDT), *Bacteroides fragilis* toxin and cytotoxic necrotizing factor-1 can damage DNA repair machinery leading to tumorigenesis (133, 139). The toxin microcystin from *Cyanobacteria* was associated with diminished CD36 and upregulation of poly ADP ribose polymerase 1 (PARP1) levels *in silico*, which was confirmed in human lung epithelial carcinoma cells (A427) *in vitro* and in human adenocarcinoma samples (134). Similarly, heat-inactivated *E. coli* stimulated TLR4 and induced NSCLC metastasis and adhesion *in vivo*. These effects were specifically induced through extracellular signal-regulated kinase (ERK)1/2 and p38 mitogen-activated protein kinases (MAPK) pathways (140).

Some bacterial species directly promote the development of lung cancer while some are found in lung cancer patients after tumor has progressed. A pooled meta-analysis study suggests that *H. pylori* promotes lung cancer progression by 3.24-fold compared to controls (without known history of *H. pylori* infection and lung cancer) (141). Another meta-analysis suggests that previous lung disease with pneumonia and *Mycobacterium tuberculosis* could significantly increase lung cancer risk (142). Similarly, chronic infections with *Chlamydia pneumoniae* are also associated with increased risk in male patients aged ≤55 years (143). D'Journo et al., investigated the association of the lung microbiome and cancer through RT-PCR of 16S rRNA genes of bacteria in lung cancer patients. Microbiological examination was carried out in lung sections of patients selected for major lung surgery. This study showed that *Cytomegalovirus* were abundant in the non-cancer distal airway and parenchyma of lung sections (144). Similarly, Lee et al., investigated the lung microbiome in the BALF samples from lung cancer patients as well as those with benign mass-like lesions. 16S rRNA sequencing of BALF samples showed that two phyla (TM7 and Firmicutes) were more abundant in lung cancer. Also, the genera *Veillonella* and *Megasphaera* were comparatively higher in lung cancer patients, suggesting that dysbiosis may be a biomarker for lung cancer (137).

The association of the commensal microbiome with development of lung cancer was further elaborated in a nested case control study of 4,336 subjects with lung cancer and 10,000 controls. This study was performed with subjects 40–84 years of age to find links between the use of antibiotics and cancer risk. The relative risk of lung cancer was 2.52 (95% CI, 2.25–2.83) in subjects receiving ≥10 antibiotics compared to controls with no antibiotics. The higher relative risk was probably because of inflammatory conditions due to frequent infection and alterations in lung microbiomes in patients taking antibiotics (145).

The lung microbiome is critical in maintaining immune homeostasis in the airway mucosa. Alterations in the normal lung microenvironment can have deleterious effects including tumor progression. An *in vivo* mouse model study showed that antibiotic treatment makes mice more prone to the formation of engrafted Lewis lung carcinoma and B16/F10 melanoma, resulting in increased tumor multiplicity and sizeable tumor foci in the lungs and reduced mean survival. Treatment resulted in the malfunction of γδT17 cell responses in the lungs, leading to more invasive tumor progression. Restoration of altered immune surveillance in antibiotic treated mice could be achieved by adding IL-17 producing γδT cells and by supplementation with IL-17. The promotion of lung cancer development by commensal bacteria is mediated by activating lung-resident γδT cells to provoke inflammation (146). This recent discovery in mice introduced a new cancer-promoting mechanism for microbiome-activated γδT17 cells in lung cancer, highlighting the complex balance between inflammation and immune surveillance in the lung. Collectively, these studies highlight the significance of commensal bacteria in strengthening the immune response and protecting against lung cancer (147).

In addition to lung cancer progression, the lung microbiome can be linked to cancer phenotype. More specifically, the distribution of bacterial species can vary between different lung cancer subtypes. *Comamonas, Rhodoferax, Acidovorax, Polarmonas*, and *Klebsiella* are often observed in squamous cell carcinoma (SCC) but not adenocarcinoma (133). Apopa et al., investigated the microbiome composition in lung squamous cell carcinoma, adenocarcinoma and healthy control tissues and found that the phylum Cyanobacteria was persistent in adenocarcinoma samples (134). The potential association between salivary microbiome and lung cancer occurrence was evaluated in subjects with lung adenocarcinoma and squamous cell carcinoma. Deep sequencing analysis of the salivary microbiome of these lung cancers were compared with healthy controls, which showed that the genera Veillonella, Capnocytophaga, Neisseria, and Selenomonas were remarkably higher in subjects with cancer compared to controls (138). A recent clinical study of 143 lung cancer patients and 33 healthy controls revealed the presence of a distinct lung microbiome in the cancer group compared to controls. Particularly, in patients with squamous cell carcinoma, *Acidovorax temporans* was detected in tumor tissues by fluorescent *in situ* hybridization. This was further confirmed with 16S rRNA analysis where *Acidovorax* dominated in squamous cell carcinoma with mutations in the TP53 gene, but was not observed in adenocarcinoma (133).

The characterization of lung microbiomes in lung cancer patients show heterogeneity among various stages of disease. Analysis of seven and 151 patients with stage IV and I-IIIA stage lung cancer, respectively, showed that the genus Thermos was more abundant in stage IV (46). Huang et al., analyzed the differential taxonomy of squamous cell carcinoma with (SCC_M1) or without metastasis (SCC_M0) and lung adenocarcinoma with (AD_M1) or without metastasis (AD_M1). In stage I-III, the phylum Firmicutes and genera Veillonella, Megasphaera, Actinomyces and Arthrobacter were increased in AD_M0 compared to SCC_M0. In stage IV, the genera Capnocytophaga and Rothia were decreased in AD_M1 compared with SCC_M1. Interestingly, no difference was observed in these 6 genera among former smoking and non-smoking groups indicating that smoking was not responsible for the variation in the differential genera (148).

Recently, researchers have began focusing on opportunities to treat lung cancer by manipulating the lung microbiome. Targeting the microbiome and their products (toxin/metabolites) could be a promising approach as adjunct therapies or alone as suggested by various *in vivo* and *in vitro* studies (149). Oral administration of *Enterococcus hirae* and *Barnesiella intestinihominis* enhanced the therapeutic efficacy of cyclophosphamide and increased the survival in lung cancer patients. Bacteria specific memory Th1 cell immune responses selectively predicted the longer progression free survival in patients with advanced lung cancer (150). Another *in vivo* study in mice evaluated the efficacy of an antitumor vaccine prepared from *B. subtilis* B-7025 in experimental models of solid sarcoma 37 (S37) and metastatic Lewis lung carcinoma. The vaccine was prepared on the basis of lectines from *B. subtilis* B-7025 and its synergetic effects tested with concurrent administration of a probiotic mixture of *Enterococcus faecium* K-50 and *Saccharomyces cerevisiae* 14K, compared to vaccine alone. Combination therapy resulted in potent synergistic anti-tumor effects in S37-bearing mice. Moreover, there was 2 to 2.5-fold inhibition of metastasis of Lewis lung carcinoma compared to vaccine alone (151). Similarly, an *in vitro* study in a lung cancer cell line (SK-MES-1) showed probiotics *Lactococcus lactis* KC24 and NK34 from Korean fermented food Kimchi resulted in 86.53 ± 0.96% and 96.71 ± 0.00% reduction in lung cancer cell viability, respectively (152, 153). Taken together, these data show that targeting the microbiome or its metabolites could be a novel way to manage lung cancer progression. However, more *in vitro* and *in vivo* studies are essential to validate and explain the evidence-based beneficial roles of probiotics in lung cancer.

## Lung Microbiome and IPF

Recently, a potential role of microbiomes in IPF has been reported (154, 155). Specifically, it was shown that changes in bacterial diversity were related to disease progression. However, how the microbiome influences respiratory function and how it can be used as a clinical marker in IPF remains to be elucidated. Some potential roles of the microbiome in the progression of IPF have been reported (156). Dysbiosis and the colonization of pathogenic bacteria can cause epithelial cell injury and activate immune responses leading to pro-inflammatory and pro-fibrotic cascades resulting in structural changes in the lungs (157). Current therapies for IPF are limited and glucocorticoids or immunosuppressive agents are associated with increased risk of death and hospitalization. Fortunately, antibiotics can improve quality of life and reduce mortality suggesting that the microbiome may have a role in the pathogenesis of the disease (158–160).

Interestingly, studies have reported that BALF from IPF patients have changes in the lung microbiome due to changes in the abundance of the phyla *Firmicutes, Proteobacteria, Bacteroidetes*, and *Actinobacteria*. Specifically, there was a decrease in diversity due to an increase in *Firmicutes* and a decrease in *Proteobacteria* (161). This loss of diversity correlated with IPF symptoms including low forced expiratory and vital capacity, high serum surfactant protein-D and lactate dehydrogenase (both clinical biomarkers of IPF progression). The decrease of lung bacterial diversity correlated with pro-inflammatory and pro-fibrotic cytokines in the alveoli in IPF patients, suggesting that loss of diversity may have some impact on pathogenesis (156).

A higher bacterial burden has been observed in patients with IPF who had minor allele at the promoter of the mucin 5B (MUC5B), a gene that encodes a mucin family of protein which composes mucus, suggesting that the bacterial burden and MUC5B mutation are linked mechanistically (154, 162–165). This suggests that MUC5B may play a key role in IPF pathogenesis. Moreover, the development and progression of IPF has been associated with single nucleotide polymorphisms in microbially linked genes such as Toll-interacting proteins, which operate as an adaptor protein for TLRs that mediate microbe-host interactions (165, 166). Some studies found that BALF neutrophilia and expression levels of genes involved in host defense response (*NLRC4, PGLYRP1, MMP9, DEFA4*) as well as encoding antibacterial peptides (*SLPI* and *CAMP*) were increased suggesting a correlation with both increased bacterial burden and disease progression in IPF (167). The absence of secretory leukocyte protease inhibitor (SLPI), a host protein implicated in IPF, leads to the impairment of collagen gene expression in a mouse model of bleomycin-induced lung fibrosis (168). This suggests that there is a potential mechanistic link between the microbiome and lung fibrosis. During IPF exacerbations, patients have increased numbers of neutrophils in BALF due to increased bacterial burden, suggesting that the microbiome might play a causative role in exacerbations (169). Overall, these studies suggest that genetics and transcriptomic alterations in innate immune response genes are all associated with increases in bacterial load in IPF and its progression. This increase in bacterial burden is associated with increased epithelial cell damage, which leads to impaired lung function (170, 171). Interestingly, study demonstrate that fibroblasts obtained from IPF patients who had an altered microbiome or increased microbial load showed enhanced expression of the α-smooth muscle actin gene, which is a marker of lung fibrosis (172). Moreover, it has been reported that lung dysbiosis induces pro-inflammatory cytokines, including IL-17B and TNF-α, that cooperatively activate lung epithelial cells leading to inflammation and fibrosis (160), suggesting that there is a strong mechanistic link between the microbiome and fibrogenesis.

Despite these studies demonstrating the association between the microbiome and fibrogenesis, key questions remain unanswered. Firstly, is microbial diversity or the abundance of specific species responsible for the development and progression of IPF. Secondly, can the lung microbiome serve as a biomarker for the development and progression of IPF. These questions remain challenging and require further study.

## Discussion and Future Perspectives

Numerous studies have shown important roles for the lung microbiome in both the maintenance of respiratory homeostasis and pathogenesis of CLDs (1, 4, 34). Thus, there is an exciting opportunity to target the lung microbiome for the treatment of CLDs. Even though microbiome modulation as a therapy for CLDs is currently limited, antibiotics, anti-inflammatory agents, probiotics and diet can help significantly in reducing disease exacerbations (33, 173, 174). Importantly, proper analysis and understanding the spectrum of the effects of the lung microbiome creates new avenues for the development of targeted therapies as well as prognostic and diagnostic markers for CLDs (34).

The universal adoption of culture independent techniques including 16s rRNA gene sequencing and now metagenomics has advanced the study of the lung microbiome in recent years. However, caution is needed when applying these techniques as laboratory contamination from reagents can impact the analysis of microbiome sequencing (175, 176). Indeed, contamination can heavily compromise the consistency of microbiome data, specifically in low microbiome biomass samples such as the lungs. A study investigating the extent of contamination attributed by consumables such as the PCR master mix and DNA extraction kits during 16S rRNA gene sequencing revealed that the master mix was a prime source. This was supported by a significantly reduced blank signal and improved precision by enzymatic removal of contamination (177). Thus, there is a need to optimize and standardize methods of DNA extraction from various sample types when sequencing the microbiome.

It is well known that there is a close relationship between the lung microbiome and inflammation and innate immune responses (178–181). LPS is a classical bacterial component that can induce inflammation in the lungs, which activates innate immune responses in humans and mice (182). LPS acts via TLR4 and induces proinflammatory signaling in COPD as well as in asthma (183–185). Other bacterial components like peptidoglycans and lipoteichoic acids in Gram-positive bacteria, act synergistically to induce lung inflammation by neutrophilic influx and by release of IL-6 (186–188). Likewise, outer membrane vesicles derived from lung commensal microbes (*Bacteroides* and *Prevotella*) can stimulate IL-17B production through TLR-MyD88 adaptor signaling to promote IPF (160).

The main therapy for asthma includes anti-inflammatory corticosteroids, which are generally administered in combination with short- or long-acting β2-adrenoceptor agonists (bronchodilators) (24, 189). Although combination therapies improve symptoms by suppressing type 2 cytokine responses, inflammation and by dilating the airways, they possess nonspecific anti-inflammatory activity and have long term adverse side effects if administered at higher doses (6). In asthma, the increased load and alterations to the composition of the lung microbiome is correlated with elevated eosinophil and neutrophil numbers in the bronchi, and IL-1β, IL-6, IL-17A, IL-8, IL-12, and TNF-α levels in sputum (190). A healthy microbiome during the early stage of life plays a key role in lung development, reducing the possibility of the development of asthma (191), providing impetus for giving probiotics and prebiotics in early life to improve the microbiome (192). Pre-clinical investigations into the therapeutic potential of pro- and prebiotics have shown that altering the microbiome can influence overall immunity and reduce allergic inflammation and sensitization, suggesting the protective effect of pre- and probiotics for asthma (193). Furthermore, macrolide-based antibiotics have been studied as a long-term treatment option to decrease airway inflammation and hypersensitiveness (194). In a landmark study by Gibson et al., azithromycin treatment led to decreased bacterial burden in the lung and gut, which reduced asthma exacerbations (195). In investigations of the lung microbiome with corticosteroid treatment, Goleva et al., showed that there are specific Gram-negative bacteria which trigger corticosteroid resistance *via* factor-b–associated kinase-1 (TAK1)/MAPK activation (196). Overall, the use of pre- and probiotic supplements, macrolide antibiotics and corticosteroids can reduce asthma exacerbations by decreasing harmful bacterial load (194).

Likewise, for IPF, various reports suggest that targeting the microbiome can increase patients' quality of life (15). In an observational pilot study in 14 advanced IPF patients, treatment with the combination of the anti-viral drug ganciclovir for 2 weeks (5 mg/kg twice daily) and prednisolone reduced disease progression in eight patients as shown by improvement in forced vital capacity (197). This study suggests that there might be a viral co-factors contributing to IPF progression that can be further validated through randomized controlled trials (197). Similarly, co-trimoxazole, a combination of trimethoprim and sulfamethoxazole bactericidal drugs, reduced respiratory infections and mortality, suggesting the use of antibiotics may be beneficial as a combination therapy along with standard drugs for IPF (198). Additionally, there are two ongoing clinical trials with antibiotic treatment in IPF patients. In one, the macrolide azithromycin is being used to treat cough and improve lung function^1^. In the second, a conjugated therapy of antibiotics co-trimoxazole and doxycycline is being tested in IPF patients^2^.

In COPD, bronchoscopy sampling from the lower respiratory tract showed that 54% of exacerbations are due to bacterial infections, which shows the potential of anti-bacterial agents (199). In a longitudinal clinical study, antibiotic treatments reduced harmful bacteria, whereas corticosteroids led to increased pathogenic bacteria levels, which clearly shows the effectiveness of antibiotic treatment in COPD (200). A randomized clinical trial conducted by Clancy et al., found that an oral non-*typeable H. influenzae* vaccine did not protect patients with COPD against exacerbations although patients were not stratified for *H. infleunzae* carriage (201). Similarly, a recent systematic review indicated that there was no reduction in the severity and frequency of acute exacerbations after oral administration of *H. influenzae* vaccine in people with chronic bronchitis and COPD (202). However, more positive results may be achieved if patients were stratified and treated according to whether they were *H. influenzae* positive. Apart from the conventional therapy with inhaled corticosteroids, long-acting muscarinic antagonist and long-acting β-agonist, antibiotics and anti-inflammatory treatment helps in overcoming COPD exacerbations. However, studies are required to define their effects on the lung microbiome (203).

Pre-clinical and clinical studies suggest that associations of the microbiome with lung cancer and further studies explaining the nature of these interactions are needed (204). Detection of specific bacteria like *Veillonella* and *Capnocytophaga* by 16S sequencing in saliva samples from lung cancer patients confirm alterations in the microbiome in lung cancer (138). Furthermore, immunotherapy with monoclonal antibodies with immune checkpoint inhibitors like programmed cell death (PD)-1 and targeting its ligand PD-L1 is used in many metastatic cancers as a therapy (205). Interestingly, the use of antibiotics in lung cancer patients resulted in reduced PD-1 response due to dysbiosis which clearly shows the role of microbiome in lung cancer (206). Until now, there are no reports of people using any specific treatments to target the microbiome during lung cancer, which may be a potential therapeutic and diagnostic tool.

**Figure 1:**
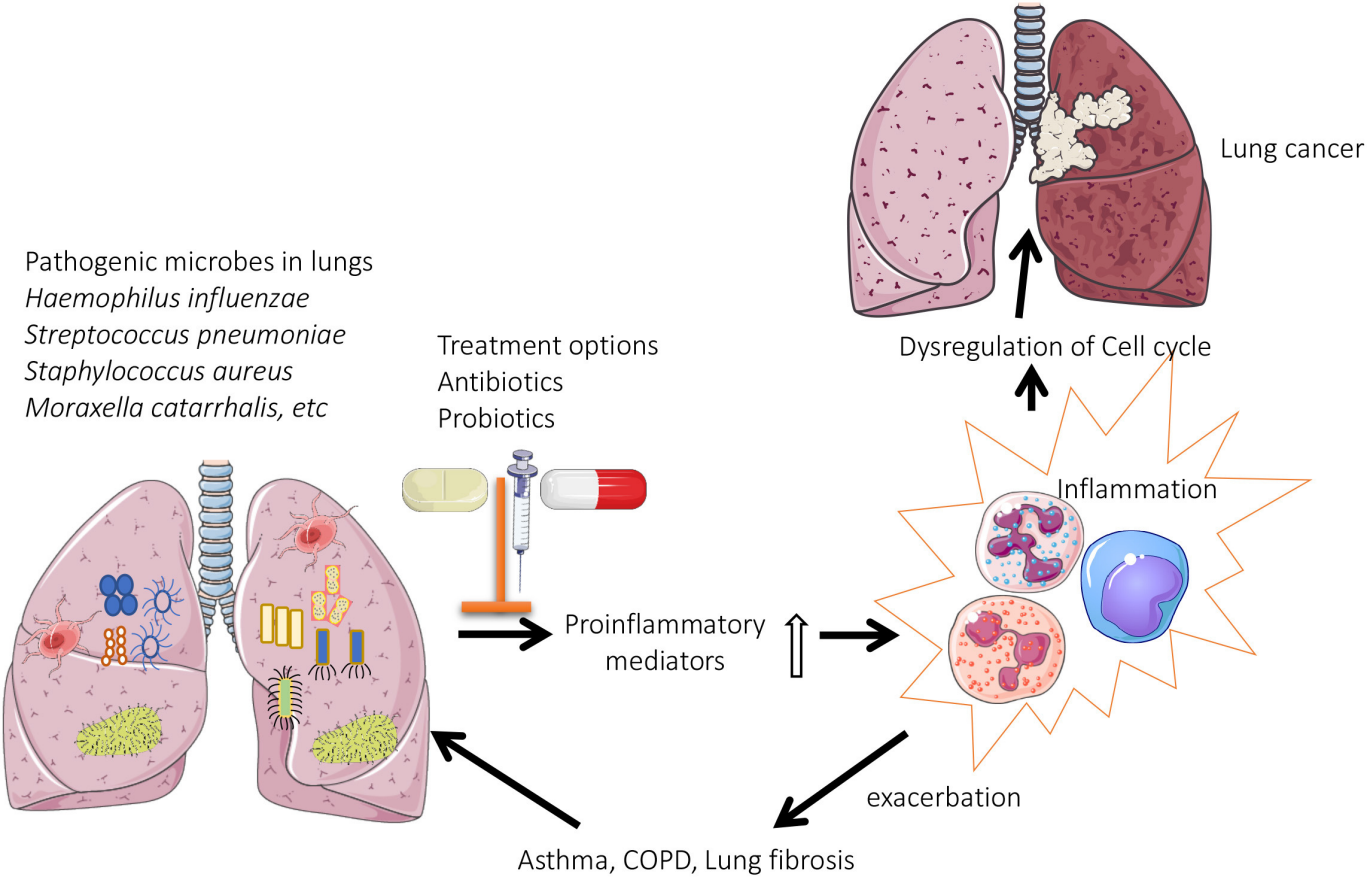
Role of lung microbiome in the pathogenesis of chronic lung diseases: Pathogenic microbes are predominately present in the lungs of patients with chronic respiratory diseases. This results in enhanced production of proinflammatory mediators, which eventually leads to oxidative stress induced dysregulation of cell cycle (lung cancer) or exacerbations associated with asthma/COPD.

Overall, targeting the lung microbiome and reverting dysbiosis may prove beneficial in CLDs. The use of probiotics and antibiotics appear beneficial (Figure 1), but robust cause and effect is lacking, and it is essential that treatment studies and translational research are performed to understand and apply therapeutic interventions. These then need to be formally tested in clinical trials and then implemented into clinical practice.

## Author Contributions

KP and VD have contributed equally to this manuscript. KP, VD, and PH design the conceptual outline of the review. KP, VD, VP, IG, RW, and VM wrote the review. SS, KB, NH, MB, KD, AV, MK-C, and IY provided critical feedback and proof-read the manuscript. All authors contributed to the article and approved the submission version.

## Conflict of Interest

The authors declare that the research was conducted in the absence of any commercial or financial relationships that could be construed as a potential conflict of interest.

## Footnotes

^1^https://clinicaltrials.gov/ct2/show/NCT02173145

^2^https://clinicaltrials.gov/ct2/show/NCT02759120
